# Biomarker Identification in Breast Cancer: Beta-Adrenergic Receptor Signaling and Pathways to Therapeutic Response

**DOI:** 10.5936/csbj.201303003

**Published:** 2013-05-21

**Authors:** Liana E. Kafetzopoulou, David J. Boocock, Gopal Krishna R. Dhondalay, Desmond G. Powe, Graham R. Ball

**Affiliations:** a The John van Geest Cancer Research Centre, School of Science and Technology, Nottingham Trent University, Nottingham, NG11 8NS, UK; b Department of Cellular Pathology, Nottingham University Hospitals Trust and University of Nottingham, Nottingham, NG7 2UH, UK

**Keywords:** Artificial Neural Networks, Microarray Data, Beta-2-Adrenergic Receptor, beta-blockers

## Abstract

Recent preclinical studies have associated beta-adrenergic receptor (β-AR) signaling with breast cancer pathways such as progression and metastasis. These findings have been supported by clinical and epidemiological studies which examined the effect of beta-blocker therapy on breast cancer metastasis, recurrence and mortality. Results from these studies have provided initial evidence for the inhibition of cell migration in breast cancer by beta-blockers and have introduced the beta-adrenergic receptor pathways as a target for therapy. This paper analyzes gene expression profiles in breast cancer patients, utilising Artificial Neural Networks (ANNs) to identify molecular signatures corresponding to possible disease management pathways and biomarker treatment strategies associated with beta-2-adrenergic receptor (ADRB2) cell signaling. The adrenergic receptor relationship to cancer is investigated in order to validate the results of recent studies that suggest the use of beta-blockers for breast cancer therapy. A panel of genes is identified which has previously been reported to play an important role in cancer and also to be involved in the beta-adrenergic receptor signaling.

## Introduction

Epidemiological studies have suggested the influence of host factors in both survival and the recurrence of breast cancer, including psychological factors such as depression and chronic stress [1, 2]. The effects are mediated through hormonal and inflammatory pathways and have been found to influence breast cancer progression, angiogenesis and metastasis [3]. Recent studies have showed the importance of the sympathetic nervous system and neuroendocrine regulation in breast cancer [4–6]. More specifically, beta-adrenergic receptor signaling has been identified to regulate cellular processes involved in cancer initiation, progression and metastasis [3, 4, 7]. As a result, research interest has focused on the positive impact that beta adrenergic-receptor antagonist drugs may have on cancer growth and metastasis [8–10].

Breast cancer is a complex disease with great heterogeneity and is one of the most common malignancies present in women, the complexity it presents arises from its different biological features and its diverse clinical outcomes [11]. Clinical parameters such as tumor grade and age, along with biomarkers currently available such as estrogen receptor (ER) and progesterone receptor (PR) status, do not provide the information to fully understand and describe the complexity of cancer [12]. This has led to the understanding that cancer has to be interrogated as a greater system of different disease types, giving rise to the need to identify new markers that will provide the ability to further categorize the different subtypes of the disease. Identification and validation of new molecular targets will allow for new potential therapies.

Identification and validation of biomarkers has proven essential in disease diagnosis, disease stage determination and personal treatment guidance [11]. Understanding the pathways involved in complex disease states, such as cancer, has proven significant in the identification of effective treatment and detection methods. Diagnostic biomarkers have resulted in great advances, such as targeting specific molecules to inhibit tumor growth, but have also highlighted limitations, since complex disease states such as cancer emerge as a result of interactions of multiple molecules and different molecular pathways.

Identification of groups of markers and an understanding of their interactions allows for greater understanding of disease pathways and the biological functions of associated genes. This complex collection of information is described by the word “interactome”, which was first defined in 1999 by Sanchez *et al*., and describes the complete group of interactions that are encoded by the genome of a specific organism, biological state or disease [13]. Understanding the interactome of cancer will allow the development of novel approaches to tackle its occurrence, progression and metastasis. Defining the interactome of an organism, biological state or disease, is a complex task and presents limitations to the approaches that can be used to analyze the genome and the interactions occurring within it [14, 15]. Thus it is necessary to assess a specific question and investigate the interactome in the concept of that question. This is an approach introduced by Lancashire *et al*., and has been used successfully to screen genes in the content of a specific question, introducing less complexity to the approach [15].

Gene expression microarrays allow for the detection of the presence and abundance of the mRNA hybridized to DNA on the array surface which ultimately provides information about the genomic profile of an organism [14]. Expression arrays are a high throughput analytical tool which allows statistical analysis of the genomic profile of an individual or a patient [16, 17]. Such an analysis allows identification of specific patterns present within the patient profiles associated with disease status and disease characteristics [18]. This information has proven vital for the identification of new treatments and for further understanding of disease pathways [19].

Over recent years, data analysis has presented significant challenges, due to the huge amount of data generated. Technologies such as microarrays present great tools for the genomic era, but the large amount of information generated and their multidimensional nature introduce limitations for data analysis [20]. The volume of medical data available and the growing need for personalized medicine and diagnosis have introduced ANNs into biomedicine with various applications in different disciplines and fields. ANNs are a form of artificial intelligence which has been shown to be capable of modeling complex data with high predictive accuracy [21]. Other advantages are that ANNs have the ability to tolerate noisy data and they are also capable of generalisation. Their importance is highlighted through their pattern recognition capabilities and due to their ability to generate reproducible and robust information.

## Experimental Procedure

A systems biology approach was followed to interrogate the adrenergic receptor system using a collection of experimental array data and ANNs as an analytical tool. The ANN approach is used to analyze a large cohort of non-linear data using a gene of interest as an input to produce a list of genes in ranking order of best prediction as an output. Transcription profiling of human breast cancer samples were used and trends in gene expressions were studied using the adrenergic gene as an input.

The EMBL-EBI database library (www.ebi.ac.uk/arrayexpress) was used to identify a suitable dataset for our analysis. The data set chosen was labeled as E-GEOD-4922. The dataset consisted of transcription profiling of 578 human breast cancer samples, from Uppsala and Singapore cohorts. The dataset samples were obtained from both A-AFFY-33 (Affymetrix GeneChip Human Genome HG-U133A) and A-AFFY-34 (Affymetrix GeneChip Human Genome HG-U133B) platforms. Clinical and pathological characteristics of the patient samples are presented in Table 1. The dataset is comprized of 578 samples of which 422 were ER+ (estrogen receptor positive) and 156 samples which were negative, unknown or blank of information. This study focuses on the ER+ cases due the significant sample size. A dataset equally as big for ER- (estrogen receptor negative) was not identified thus a comparable study was not possible. Samples that were characterized as negative, unknown or blank of information for estrogen receptor status, were excluded from the analysis. This led to a total of 422 samples that were further processed to compile the information of each patient within one file. The final file contained 211 patient profiles, including both information from A-AFFY-33 (22,283 genes) and A-AFFY-34 (22,645 genes). Each patient profile is associated with 44,928 gene probes.

**Table 1:** Clinical and pathological characteristics of dataset used.

Data Information	Patient Sample Number/Information	Percentage
**Age (years) at diagnosis**		
Mean	62.1	
Median	63.02	
Age range	28-93	
**Tumor Size:**		
Mean	2.24 cm	
Tumor size range	0.2-13.0 cm	
T1 a + b (≤1.0 cm)	22	8.80%
T1 c (>1.0 cm-2.0 cm)	104	41.80%
T2 (>2.0 cm-5 cm)	117	47.00%
T3 (>5 cm)	6	2.40%
**Lymph node involvement**		
No	159	66.30%
Yes	81	33.70%
**Grade:**		
1	68	27.30%
2	126	50.60%
3	55	22.10%
**Estrogen receptor (ER)**		
Negative	34	13.90%
Positive	211	86.10%
**Recurrence**		
No	160	64.30%
Yes	89	35.70%
**Survival in months**		
Mean	85.7	
Median	119	
Survival range	0-153	
Alive	160	64.30%
Dead	89	35.70%
**Hormone therapy (Tamoxifen)**		
No	183	73.50%
Yes	66	26.50%
**Chemotherapy**		
No	208	83.50%
Yes (CMF)	41	16.50%

The microarray data was analyzed using the ANN stepwise method, which incorporates a three-layer feed-forward multi-layer perceptron (MLP) with a back propagation (BP) algorithm and a sigmoidal transfer function. Learning rate and momentum were set to 0.1 and 0.5 respectively. The algorithm incorporates two hidden nodes (to maintain a parsimonious solution) in the hidden layer and utilizes a Monte Carlo cross-validation (MCCV) and a bootstrapping approach, which is used to provide an unbiased estimation of the error rate. MCCV randomly assigns training, validation and test sets which in this case include 60%, 20% and 20% respectively [20, 22, 23]. All three groups are assigned the cases randomly. Bootstrapping is used due to its reliability for generalisation of the network. The training subset includes 127 patient profiles (60%), the test subset includes 42 patient profiles (20%) and the validation subset also includes 42 patient profiles (20%). The test subset allows the model to be independently tested on a blind data set and the validation subset assesses the model performance during the training process [14, 24].

Each stepwise analysis generated 5 files, one for each loop it was set to run. A file was then created containing the averaged information, which was arranged in order of ascending average test error. The input probes were examined using the median training performance (percentage of correctly classified cases) and their average test Root Mean Squared (RMS) error. The top 100 probes were selected from the list (RMS error <0.12, Figure 2) resulting in the most important genes being utilized for further study.

**Figure 1:**
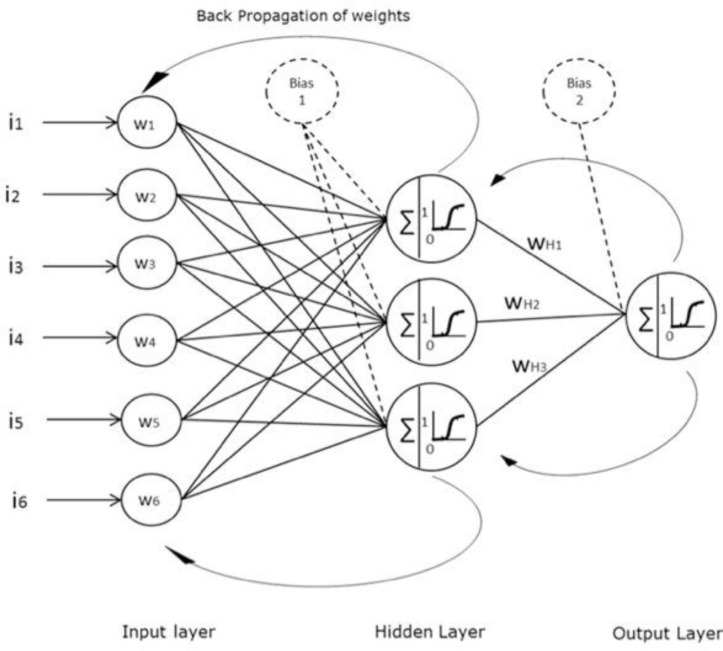
Artificial Neural Network design used for the specific project. Inputs (i_x_) are fed into the algorithm and adjusted with a corresponding weight (w_x_), and then summed and processed using a sigmoidal function, and a bias input. Output is adjusted to weights (w_HX_), summed and fitted to the sigmoidal function. Through each step the back propagation algorithm is used to adjust the weights and improve the performance.

**Figure 2:**
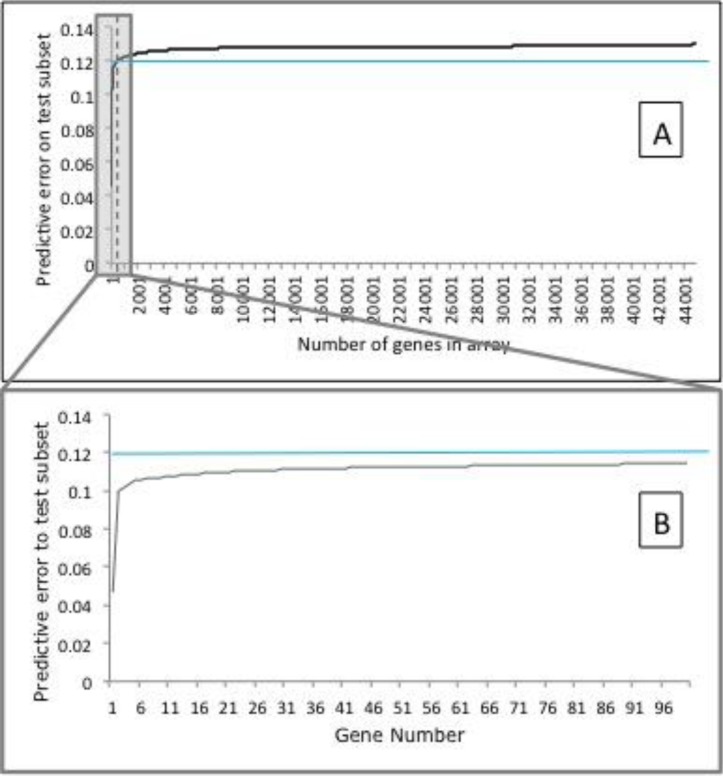
(A) Predictive error distribution of the genes for unseen data. The dashed line indicates the position of the 100^th^ gene. The blue line is included to identify the increase of the predictive error value. (B) Predictive error distribution of the top 100 genes for unseen data. Blue line is included to indicate the increase of the predictive error value. (A, B) Graph B is a zoomed section of A, to show the reason the cut-off value was selected at the top 100 probes. The blue line in both graphs is set at 0.12 which shows the increase of the predictive error after the top 100 probes. Error bars are not included in both graphs for clarity.

After analysing the first round of data it was concluded that the analysis would focus on building a map with beta-2-adrenergic receptor as the initial starting point. A non-reductionist network growth approach was used as an analysis strategy. ADRB2 was used to create a network of important genes and to study links between them. All the data was generated and the results were studied and analyzed conducting network inference. A simplistic network was created for the input probe and the top 10 interconnections were identified for the first set of data and presented in that network. The results were studied in general to identify commonalities between the probe sets and also to identify patterns within the data.

## Results

The probe corresponding to ADRB2 was identified and used as the input for the analysis. The top 100 ranking probes were studied and the top 10 ranking genes were analyzed further due to their good performance (based on their low predictive error value). Figure 2 explains the selection process and the reason the top 100 probes were used as the cut off value.

ADRB2 was the initial input gene of the analysis and the top 10 ranking genes were further analyzed to identify patterns within the data and common gene signatures. As seen in Table 2, the first ranking probe corresponds to the *ADRB2* gene, this allows for validation of the technique, since it informs us that the probe is the most predictive for itself. The genes following are the top 10 most predictive genes for *ADRB2* gene expression. The genes identified are listed in Table 2 and their gene names are listed in Table 3.

**Table 2:** Summary of top ranked 11 genes from stepwise analysis of the beta-2-adrenergic receptor gene. Numbers of interconnections with other genes have been included from the analysis of each of the top 10 genes identified.

Rank order	Corresponding Gene Symbol	Probe ID	No. of interconnections with other genes
1	*ADRB2*	206170_at	10
2	*DARC*	208335_s_at	8
3	*ENPP2*	209392_at, 210839_s_at	7
4	*ABI3BP*	223395_at	7
5	*HS2ST1*	215039_at	6
6	*CHRDL1*	209763_at	7
7	*SCARA5*	229839_at	7
8	*SELP*	206049_at	6
9	*MFNG*	204153_s_at	3
10	*ITIH5*	219064_at	4
11	*CD69*	209795_at	1

**Table 3:** Top 10 genes identified and their gene names.

Gene Symbol	Gene name
ADRB2	Adrenoreceptor beta 2, surface
DARC	Duffy blood group, chemokine receptor
ENPP2	Ectonucleotide pyrophosphatase/ phosphodiesterase 2
ABI3BP	ABI family, member 3 binding protein
HS2ST1	Heparan sulfate 2-O-sulfotransferase
CHRDL1	Chordin-like 1
SCARA5	Scavenger receptor class A, member 5 (putative)
SELP	Selectin P
MFNG	MFNG O-fucodylpeptide 3-beta-N-acetylglucosaminyltransferase
ITIH5	Inter-alpha-trypsin inhibitor heavy chain family, member 5
CD69	CD69 molecule

**Figure 3:**
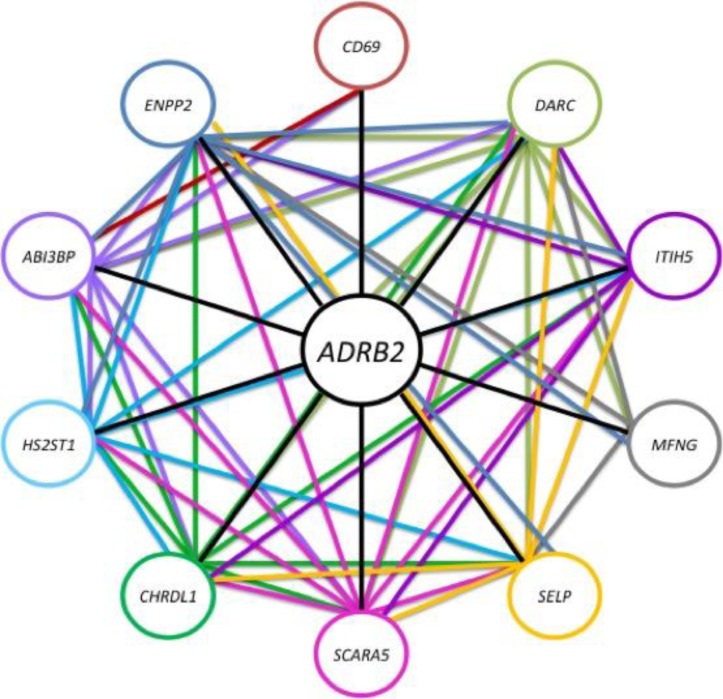
ADRB2 network created presenting the top 10 genes resulting from its analysis and interconnections occurring in second layer analysis. Connections are color coded and correspond to the equivalent colored gene. Connections with the same color have originated from the analysis of the common equivalently colored gene.

A simplistic network has been constructed which presents the exact number of interconnections occurring in the further analysis of the top 10 genes. The interconnections can be seen both in Table 2 and Figure 3 which presents the analysis technique along with the interesting aspect of our results since there are multiple connections occurring between the genes identified.

By studying the top 100 probes for each of the top 11 genes analyzed it was possible to identify common genes and patterns occurring within the data. Table 4 presents the most common gene signatures. Genes from Table 4 were selected for further analysis and the results were compared with the data obtained from the analysis of ADRB2 and its top 10 genes. The large amount of data generated from the analysis of all the genes selected did not allow for an extensive analysis, but gave the opportunity to validate the results obtained from the previous runs. The data was studied and most genes found from the analysis of ADRB2 and the top 10 genes were found to reoccur. Tables 5 and 6 present important immunologically related genes and important cancer related genes which were identified.

**Table 4:** List of the most frequently occurring genes (in 10 runs). Genes presented are genes with a large amount of cross linkage and therefore associated in the pathway.

Gene Symbol	Gene Probe	Frequency (/10)	Details
*ABCA8*	204719_at_A	9	Single probe
*ANK2*	202920_at_A	9	Single probe
*C7*	202992_at_A	10	Single probe
*CAV1*	212097_at_A	7	Multiple probes
	203065_s_at_A		
*EBF1*	232204_at_B	8	Multiple probes
	227646_at_B		
*FHL1*	201540_at_A	7	Multiple probes
	201539_s_at_A		
	210299_s_at_A		
	214505_s_at_A		
	210298_x_at_A		
*IGF1*	209541_at_A	5	Multiple probes
	209540_at_A		
	209542_x_at_A		
*IL33*	209821_at_A	9	Single probe
*JAM2*	229127_at_B	8	Multiple probes
	219213_at_A		
*MEOX1*	205619_s_at_A	9	Single probe
*TNXB*	206093_x_at_A	9	Multiple probes
	213451_x_at_A		
	216333_x_at_A		
	208609_s_at_A		
*TSPAN7*	202242_at_A	8	Single probe

**Table 5:** Significant gene signatures due to strong connection with cancer found within the data.

Oncogenes	Description	Cancer related genes	Description
*MYH11*	Myosin	*RHOJ*	Ras homolog gene family, member J
*LCK*	Lymphocyte-specific protein tyrosine kinase	*FYN*	FYN oncogene related to SRC, FGR, YES
*ERG*	Member of erythroblast transformation-specific transcriptional regulator	*LYN*	v-yes-1 Yamaguchi sarcoma viral related oncogene homolog
*ETS1*	Member of erythroblast transformation-specific family of transcription factors	*PTPRC*	Protein tyrosine phosphatase, receptor, C
*FOSB*	FBJ murine osteoblastoma viral oncogene homolog B	*IGF1*	Insulin-like growth factor 1

**Table 6:** Frequently occurring immunologically related genes.

Gene Name	Description
*DARC*	Duffy blood group, chemokine receptor
*IL33*	Interleukin 33
*CXCL12*	Chemokine(C-X-C motif) ligand 12
*CCL5*	Chemokine(C-C motif) ligand 5
*CCL19*	Chemokine(C-C motif) ligand 19
*CCL21*	Chemokine(C-C motif) ligand 21
*PTPRC*	Protein tyrosine phosphatase, receptor type, C
*EBF1*	Early B-cell factor 1
*EBF3*	Early B-cell factor 3
*HLA-E*	Major histocompatibility complex, class I, E
*HLA-DMB*	Major histocompatibility complex, class II, DM beta

## Discussion

Several of the markers identified have been found to be of importance and relevance to breast cancer. Our aim was to study a cohort of breast cancer gene expression microarrays in the concept of the adrenergic receptor gene. The gene signatures found are the most predictive and of greatest relevance to the beta-2-adrenergic receptor and have been identified through an analysis of breast cancer samples. This provides information about the expression of genes both related to the adrenergic receptor as well as breast cancer.

### DARC and chemokines identified to be of importance in the adrenergic receptor system

Chemokines are chemotactic cytokines with the ability to bind to GPCRs [25]. Chemokines were initially identified as small molecules that function as activation and recruitment molecules for leukocytes such as neutrophils and monocytes; they were originally considered as mediators of inflammatory pathways [26, 27]. Chemokines and their receptors have since been discovered to have an essential role in tumor initiation, promotion and progression.

Lazennec *et al*., [26] published a review on chemokines and chemokine receptors and their involvement in cancer. They report the importance of the tumor microenvironment and that chemokines are produced by tumor cells and by cells of the tumor microenvironment such as cancer-associated fibroblasts, mesenchymal stem cells, endothelial cells, tumor-associated macrophages and tumor-associated neutrophils. The review concentrates on tumor metastasis, focusing on the concentration of chemokines produced at sites of metastasis, which attracts the cancer cells and causes them to metastasise [26]. This is one of the reasons that explain the preferential pattern occurring in metastatic sites arising from different types of cancer. The importance of *CXCL12, DARC, CCL21, and CCL5* is highlighted, which are also gene signatures arising in our analysis. The review reports their importance in tumor metastasis and the tumor microenvironment and offers various examples in the literature were they have been found to be associated with breast cancer.

### Beta-2-Adrenergic Receptor and Cancer

The beta-2-adrenergic receptor has been identified to regulate several cellular pathways and has also been found to have an important role in initiation and progression of cancer [1, 6, 28, 29]. It has been described to contribute to pathways of inflammation, angiogenesis, epithelial mesenchymal transition and apoptosis [4]. Within the tumor microenvironment and cancer pathways tumor associated macrophages have been identified to be related to the beta-adrenergic signalling pathways [4]. Powe *et al*., [9] showed that cell migration is mediated by beta-adrenergic receptors and that beta-blockers inhibited the process, specifically the antagonist propranolol. Sloan *et al*., [30] published their results on the effect of stress on metastasis development. They report that the sympathetic nervous system induces a metastatic switch in primary breast cancer and emphasize the activation of the sympathetic nervous system as a target for regulation of breast cancer metastasis. Both Powe et al. [9] and Sloan et al. [30] report the evidence and form the hypothesis of utilising the beta-adrenergic receptor for novel antimetastatic therapies that will increase survival and induce prometastatic gene expression in primary breast cancers. Cole *et al*., [4] report several pathways that have been identified to be involved both with beta-2-adrenergic receptor and cancer, specifically cellular and molecular processes that mediate beta-adrenergic receptor and its influence on tumor progression. Pathways that are mediated by the beta-adrenergic receptor include recruitment of macrophages into the tumor, increase in cytokine expression, angiogenesis, matrix metalloproteinase concentration increase in invasion, tumor cell mobilisation and motility, focal adhesion kinase mediated resistance to apoptosis, and BAD-mediated resistance to apoptosis [4]. All these pathways are of great importance in cancer and their association needs to be further investigated to conclude on the hypothesis the adrenergic receptor has an important role in breast cancer.

This study's findings, along with the studies mentioned above reveal commonalities in gene signatures that have been stated to be related to both beta-adrenergic receptor and cancer. Gene signatures such as *IL6, MMP9, MMP1, IFNGR1, CXCL12, FOSB, LCK, CCL21, DARC, ERG, MYH11,RHOJ, IGF1, ETS1* which have been identified in our research are present both in cancer pathways and beta-adrenergic pathways. It is possible to identify commonalities and also to find the genes identified in our analysis that play an important role in these pathways. This provides validation for the technique used and also gives information about the relationships between gene expression levels of cancer related genes and the adrenergic receptor. This knowledge could be used in the design of novel therapeutic strategies involving combination therapy to target upstream and downstream molecules in adrenergic receptor-mediated disease.

## Conclusions

This study provides an insight into the relationship between the beta-2-adrenergic receptor and breast cancer disease pathways. Gene signatures were identified and patterns within the results were found that correlate with the information currently available in the literature. This allows the understanding of the common pathways between the adrenergic receptor and breast cancer and provides markers which support the studies suggesting beta-blockers could be incorporated in designing new breast cancer treatment strategies. The results are promising and will be further validated to obtain greater understanding of the mechanisms they are involved in.

## Future Work

The analysis conducted has generated a large amount of data that needs to be further analyzed to investigate all the possible patterns and gene interaction that could arise. Further analysis would provide information on key pathways and possible gene associations.

The current project analyzed a single dataset, which included 211 patient samples run on two affymetrix platforms and only ER+ patients samples were included. It would be possible to expand the possibilities and design the experiment differently in order to obtain more information about various other situations. Analysis of multiple datasets would allow for comparison of the results and identification of common patterns. It would also be possible to find datasets that include an ER- cohort, or healthy donors. This would allow comparison of the results to identify different patterns occurring in different breast cancer subtypes.

Combination of other analytical techniques could also be used thus providing more confidence in the information obtained. Both *in silico* and *in vitro* techniques could be investigated and a different experimental approach could be designed in order to investigate the same cohort and compare the results. The investigation of the immunohistochemical protein expression of certain markers identified would allow for validation of the results obtained and would provide information on whether they can be used as biomarkers for breast cancer patient sample classification.
